# What guides back pain care? A content analysis of low back pain directives in the Australian context

**DOI:** 10.1186/s12961-023-00997-5

**Published:** 2023-06-13

**Authors:** Sarika Parambath, Nathalia Costa, Carmen Huckel Schneider, Fiona M. Blyth

**Affiliations:** 1 Menzies Centre for Health Policy, Faculty of Medicine and Health, University of Sydney, Sydney, Australia; 2 Sydney School of Public Health, Faculty of Medicine and Health, University of Sydney, Sydney, Australia

**Keywords:** Content analysis, Low back pain care, Policy directives

## Abstract

**Background:**

Low back pain (LBP) is a major cause of disease burden around the world. There is known clinical variation in how LBP is treated and addressed; with one cited reason the lack of availability, or use of, evidence-based guidance for clinicians, consumers, and administrators. Despite this a considerable number of policy directives such as clinical practice guidelines, models of care and clinical tools with the aim of improving quality of LBP care do exist. Here we report on the development of a repository of LBP directives developed in the Australian health system and a content analysis of those directives aimed at deepening our understanding of the guidance landscape. Specifically, we sought to determine: (1) What is the type, scale, and scope of LBP directives available? (2) Who are the key stakeholders that drive low back pain care through directives? (3) What content do they cover? (4) What are their gaps and deficiencies?

**Methods:**

We used online web search and snowballing methods to collate a repository of LBP policy documents collectively called 'directives' including Models of Care (MOC), information sheets, clinical tools, guidelines, surveys, and reports, from the last 20 years. The texts of the directives were analysed using inductive qualitative content analysis adopting methods from descriptive policy content analysis to categorise and analyse content to determine origins, actors, and themes.

**Results:**

Eighty-four directives were included in our analysis. Of those, 55 were information sheets aimed at either healthcare providers or patients, nine were clinical tools, three were reports, four were guidelines, four were MOC, two were questionnaires and five were referral forms/criteria. The three main categories of content found in the directives were 1. Low back pain features 2. Standards for clinical encounters and 3. Management of LBP, each of which gave rise to different themes and subthemes. Universities, not-for-profit organizations, government organisations, hospitals/Local Health Districts, professional organisations, consumers, and health care insurers were all involved in the production of policy directives. However, there were no clear patterns of roles, responsibilities or authority between these stakeholder groups.

**Conclusion:**

Directives have the potential to inform practice and to contribute to reducing evidence-policy-practice discordance. Documents in our repository demonstrate that while a range of directives exist across Australia, but the evidence base for many was not apparent. Qualitative content analysis of the directives showed that while there has been increasing attention given to models of care, this is not yet reflected in directives, which generally focus on more specific elements of LBP care at the individual patient and practitioner level. The sheer number and variety of directives, from a wide range of sources and various locations within the Australian health system suggests a fragmented policy landscape without clear authoritative sources. There is a need for clearer, easily accessible trustworthy policy directives that are regularly reviewed and that meet the needs of care providers, and information websites need to be evaluated regularly for their evidence-based nature and quality.

**Supplementary Information:**

The online version contains supplementary material available at 10.1186/s12961-023-00997-5.

## Introduction

### Burden of LBP

According to the Global Burden of Disease Study 2019, low back pain (LBP) was fourth among the top ten causes for Disability Adjusted Life Years (DALYs) for 25–49-year age groups and was one among the top ten causes for 10–24- and 50–74-year age groups [1]. LBP was the leading cause of Years lived with a Disability (YLD) [2, 3] and one of the major causes of disability in most high-income countries.

YLD due to musculoskeletal disorders (mainly back pain) are more than double the expected values for Australia, according to the GBD study 2016 [4], in which deviations of YLD from expected levels based on Socio-demographic Index were estimated. Through their impact on labour force participation, the economic impact of spinal disorders is enormous, as reflected by an Australian study, which reported a loss of AU $4.8 billion in individual annual earnings, AU$2.9 billion in GDP, AU$622 million in additional welfare payments and AU$ 497 million in taxation revenue for governments during the year 2009 [5]. Musculoskeletal disorders accounted for the highest estimated spending on health in 2018–2019 (AUD 14 billion or 10.3% of total health expenditure) of which, back pain constituted a significant share. [6]

### Health system context of LBP

Although LBP imposes significant challenges on both health and social systems, little has been done to overcome these challenges at a systems level [3, 7]. Two important and co-existing issues are evidence-practice-policy discordance in LBP management and lack of, or ineffective use of resources. The latter issue stems from the use of low-value care, adding to the LBP burden through lack of resourcing, resource drainage, income and health system disparities among countries, and prioritization of communicable diseases [3]. Disparities are also identified in LBP care between rural areas and cities of Australian states [8]. Previous studies examining barriers to improved LBP care indicate a need for improved governance, empowered and informed care providers, well-funded evidence-based treatment options through funding redistribution, and avoidance of harmful vested interests [7]. This is considered particularly important in complex health systems such as Australia, where the health care is provided in a mix of public and private settings and policy responsibilities are dispersed across federal, state and local levels, as well as across professional and regulatory authorities.

There has been a particular focus around reducing low-value care in LBP management due to an increase in global low-value LBP care [7]. As a contributing factor towards low-value care, Australian studies have reported discordance with guideline recommendations, due to the various challenges faced by healthcare providers such as miscommunication, difficulty meeting patient expectations and clinical judgement restriction. [2, 9]

### Directives in LBP care

For the purpose of this study, the term ' directive' will be used to refer to any policy document developed to aid back pain care. It is acknowledged that knowledge translation from evidence into policy and practice concerning LBP care is unsatisfactory [2, 3]. The ability of policy directives such as clinical practice guidelines to improve the quality of decisions and thereby quality of treatment provided by healthcare providers has been studied in reviews [10].

Models Of Care (MOC), a type of directive (defined in Table 1), are considered critical components and essential to improving musculoskeletal care [11]. Briggs et al. described and delineated the differences between MOCs and clinical practice guidelines in musculoskeletal health, both of which complement each other in care delivery. Briggs et al. also reinforced the importance of a multidisciplinary approach to MOC development in musculoskeletal care [12]. In Australia, clinical networks/health networks, the ACI (Agency for Clinical Innovation), Ministries of Health, and Chief Medical Officers constitute the different hierarchy levels of MOC development and implementation [13]. Clinical networks considered the cornerstone of health delivery, can implement organisational changes in healthcare, incorporate evidence-based changes to patient management systems and improve quality of health services [14], all possible through MOC development and implementation. Clinical networks or health networks aim at patient centred care delivery through gold-standard practices, by bringing together different stakeholders including clinicians, patients, policy makers, to improve care delivery through best value practices.

**Table 1:** Types of directives in the repository

Type of directive	Definition of directive type	Number of directives
Clinical practice guideline (CPG)	“Systematically developed statements to assist practitioner and patient decisions about appropriate health for specific clinical circumstances” (Institute of Medicine, Washington, USA) [16]	4
Model of care (MOC)	“Broadly defines the way health services are delivered, it outlines best practice care and services for a person, population group or patient cohort as they progress through the stages of a condition, injury or event” (ACI, Australia) [17]	4
Clinical tools	“Aim to synthesise all available evidence for major clinical topics for health care workers when providing patient care” (University Health Network, Canada) [18]	9
Information sheet	“Are short documents that provides basic information on a specific topic in an easy and quick-to-read format” (Center for rural health, University of North Dakota, USA) [19]	56
Surveys/questionnaires	Are products of research methods which were designed for data collection, from a defined group of audience to collect information on a specific area of interest	2
Report	“A specific form of writing that is organized around concisely identifying and examining issues, events, or findings that have happened in a physical sense, such as events that have occurred withing an organization” (Massey University, University of New Zealand) [20]	5
Referral form	A referral form is a request from one health professional to another, for the purpose of diagnosing or treating a particular health condition	6

Within this context, a content analysis of these directives can provide insights about the number of Australian LBP policies and their content, to inform practice and eventually reduce evidence-policy-practice discordance. The overarching goal is to understand the landscape of policy directives for LBP—including policies, clinical guidelines, clinical tools, MOC and information resources, to determine their types, content, and adequacy. We sought to develop a thick description of the LBP policy landscape in Australia and determine: (1) What is the type, scale, and scope of LBP directives available? (2) Who are the key stakeholders that drive low back pain care through directives? (3) What content do they cover? (4) What are their gaps and deficiencies?

## Method

This work is a part of larger project that seeks to identify system factors that drive low value care for LBP in Australia. For the purpose of this study, the term ' directive' will be used to refer to any policy document developed to aid back pain care. We adopted methods from descriptive policy content analysis [15], wherein the data from diverse types of documents collectively called 'directives' were analysed to determine their origins and content, (i.e., key themes). Directives include MOC, information sheets, clinical tools, guidelines, and reports.

### Document selection

Data for the content analysis was obtained from the directives gathered from a purpose-built repository, created as part of the ANZBACK-The Low Back Pain Centre for Research Excellence (CRE). This is the first research reporting on the development of repository.

The repository was populated by undertaking a system-wide search with broad parameters, gathering any document that included information or guidance on decision making around prevention, treatment, management, rehabilitation, and recovery from LBP. We sought to gather as many examples as possible from a wide range of sources such as public and private providers, and range of organisations, hospitals, state organisations, hospitals, state governments, professional associations. Directives were collected using three main strategies: (i) a comprehensive desktop search for Australian websites through Google, using a combination of search terms—(policy OR strategy OR guideline OR directive OR model of care OR action plan OR framework OR strategic framework) AND (back pain OR spinal pain) These terms were chosen for being the most common terms used to describe diverse types of policy documents. The websites appearing on online search included government and other organisational websites, such as workplace regulators. The same strategy was used for all states, territories, and national-level policies in Australia; (ii) an email request sent to members of the CRE and their networks; (iii) Snowballing approach-where the references and links cited in the documents collected through the two above strategies, were searched. The documents were categorized according to their type (e.g., MOC, guidelines, clinical tools, information sheets and reports) and location (state or territory).

The criteria for inclusion of directives were:

An Australian policy directive which is a MOC, guideline, information sheet, report, questionnaire, survey, or clinical tool; (i) A directive produced or endorsed by the Government, private organisations, professional organisations, or universities; (ii) A directive published in the last 20 years; (iii) A directive focussed on LBP, rather than on musculoskeletal pain or chronic pain in general. Directives that did not meet these criteria were excluded. Surveys and referral forms were added to the list of directives, for their purpose to aid back pain management. The screening was conducted by two authors, (SP and NC) where both authors screened all citations. No citation screening platforms were used.

### Data analysis

Descriptive data (author, target audience and year (when available)) were summarised and tabulated using Microsoft Excel. The text of each directive was analysed using inductive qualitative content analysis [15]. and involved the following steps: (1) SP and NC undertook open coding to derive the codes, through reading the directives multiple times; (2) codes were then grouped into categories, themes, and subthemes; (3) the emerging coding structure was discussed through team meetings and refined with all authors. During this process, we decided that if a text excerpt was related to two or more codes, it could be coded into more than one theme or subtheme. For certain themes and subthemes, quotes from the directives were used to illustrate the meaning of codes. We followed a descriptive analysis method coding the literal meanings found in the texts. This aligns with our aim of identifying the content and key messages provided by directives targeting LBP management.

## Results

### Type, scale, and scope of LBP directives available

Eighty-four directives were included in our analysis (documented in Additional file 1). Of those, fifty-six were information sheets aimed at either healthcare providers or patients, nine were clinical tools, five were reports, four were guidelines, four were MOC, two were questionnaires and six were referral forms/criteria (See Table 1). The number of directives varied between different states.

The most common type of directive found was “Information Sheets”. These documents are 1–2 pages long, and targeted at specific audiences, such as consumers, or clinicians for use in consultation. They contain information if brief formats targeted at specific situations for a specific audience and point of time. In contrast, we only found two Models of Care that were in the range of 30–60 pages.

We found fourteen directives that were published in New South Wales (NSW), thirteen in South Australia (SA), ten in Victoria, seven in Queensland, five in Western Australia (WA) and four in Tasmania. All other directives were published by Australia-wide organizations for use across the country, and not from a particular state. Among the information sheets, NSW had the most (eleven directives), Victoria had eight, South Australia had five, WA and Queensland had three and two, respectively. SA had the greatest number of clinical tools (seven), the majority produced in collaboration with Royal Adelaide Hospital.

### Content covered in the directives

**Fig. 1:**
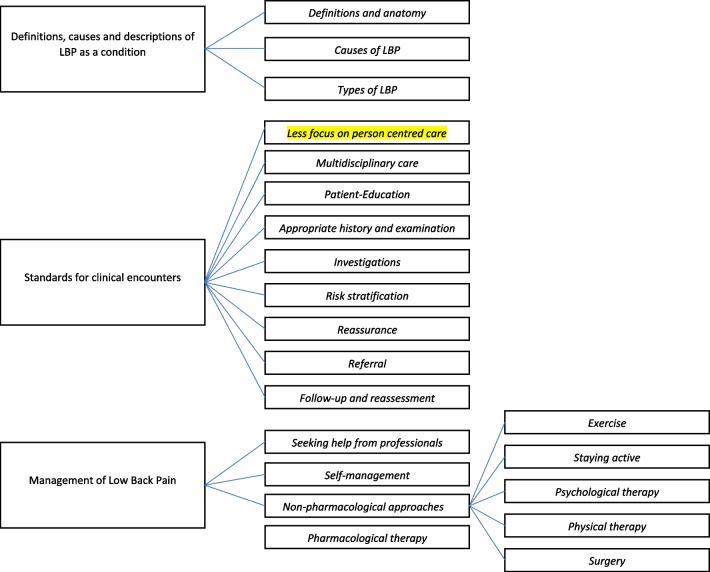
Categories, themes, and subthemes

The categories, themes, and subthemes (Fig. 1) that describe the content of the directives included in our analysis are discussed below, along with excerpts from directives that illustrate them (Additional file 2).

The three broad categories of information that were identified are 1. Definitions, causes and descriptions of LBP as a condition 2. Standards for clinical encounters and 3. Management of LBP.

Directives are referred to according to their indicator in Additional file 1 (D-1, D-2 etc.).

#### Category: Definitions, causes and descriptions of LBP as a condition

Many directives described LBP and included details on LBP definitions, anatomy, and types of LBP.

##### Themes

*Definitions and anatomy*—Some of the directives defined LBP as “Low back pain is pain that is felt in the lower part of the spine” (D-10). Such definitions were often accompanied by information about the anatomy of the spine, including the spinal cord and associated structures (D-3). Some directives use diagrams to serve the purpose.
*2)Causes of LBP—*Directives often discussed a range of factors that could cause LBP, ranging from diseases such as arthritis or osteoporosis to other causes such as stress, sciatica, and structural problems. Likewise, lifestyle factors such as day-to-day strains, physical inactivity and being overweight were discussed as common causes of LBP (D-13).
*Types of LBP—*Some directives listed and explained the types of LBP. The two main types of LBP described in the directives are acute/chronic and specific/ non-specific types (D-14).

#### Category: Standards for clinical encounters

Many of the directives, ranging from patient information sheets and clinical tools to MOC, contained information on the optimal steps to be followed while providing care to individuals with LBP.

##### Themes

1. *Less focus on person-centred care*—Only a few directives referred to or implied person-centred care in their content. These directives used terms such as 'Personalised approach', 'client needs', ‘individual patient’ and 'tailored approach' but these terms were often used without further elaboration or definition (e.g., D-9, D-32, D-11). One directive elaborated on the idea that levels of “normal” activity vary depending on what people do for a living (D-14). Likewise, one directive targeting clinicians emphasised the importance of considering individuals’ preferences (D-1).
2. *Multidisciplinary care*—The directives highlighted the importance of multidisciplinary care for people who experience persistent LBP, which was discussed as a type of LBP that requires attention from different healthcare professionals for longer periods of time. Multidisciplinary care/rehabilitation was also recommended for people at risk of developing persistent LBP. The directives also discussed how the different disciplines involved are and can be coordinated in care provision (D-32). The range of disciplines constituting multidisciplinary care was also described in some directives (D-13).
3. *Patient education*—Most directives seemed to consider patient education as a vital component of LBP management and discussed self-management as an essential aspect of LBP care (D-12). Directives often contained information on educating patients about exercise, medications, different treatment options and lifestyle changes (D-38). The significance of patient education is discussed in some directives (D-6).
4. *Appropriate history and examination*—The directives described history taking and examination as the cornerstone of patient consultation (D-37). Some portrayed it as essential to understand risk factors and decide on an imaging, referral, and treatment plan (D- 41). The information sheets for healthcare providers described the steps and procedures involved in patient examination, which were often described through flowcharts.
5. *Investigations (e.g., imaging)*—Cautious use of imaging has become a frequent theme in patient information sheets and clinical tools for care providers. Most of the directives listed the different investigation modalities available to assess LBP—which mainly constitute imaging options like X rays, CT, MRI, and bone scans. Some directives advise care providers to delay unnecessary imaging while others focus on discouraging patients from asking for unnecessary scans, with evidence including statistics (D-9). Most of the directives try to discourage patients and care providers from opting for unnecessary imaging, citing reasons such as the risks associated with radiation, unnecessary costs, and their lack of specificity (D-21).
6. *Risk stratification*—The importance of triaging and screening to reduce unnecessary imaging and guide further investigations, treatment, or referral is highlighted. Many directives ranging from MOC to information sheets provide details on the red flag and yellow flag signs and risk stratification tools. Some directives stress the importance of considering red flags in history taking and examination, and others portray it as essential to understand risk factors and decide on imaging, referral, and treatment plan. The directives list and explain the importance of red flag signs signalling serious conditions, indicating investigations or referral and yellow flag signs about psychosocial risk factors for chronicity.
7. *Reassurance*—The directives targeted at patients informed them about what to expect from the health expert, using a tone of reassurance (D-10). Reassurance was often linked to the idea of favourable prognosis and the unlikelihood of serious pathology (D-20). Some other directives reassured and encouraged patients to exercise and practice self-management (D-10). Clinical tools also advised providers to take an empathetic and reassuring approach (D-1).
8. *Referral—*The directives also highlighted the importance of timely referral to specialist or rehabilitation services. Some directives mentioned the criteria for timely referral (D-38), others did not. Directives advised care providers to identify symptoms and signs requiring early referral and to follow the existing pathways for referral (D-82). They are guided through referral criteria, eligibility protocols, communication protocols and assessment manuals. Referral was highlighted as an important part of providing multidisciplinary care (D-81).
9. *Follow up and reassessment*—Follow-up was also highlighted for the purpose of reminding and reinforcing the techniques and plans decided upon initially (D-8). Predetermined and regular follow-up was also highlighted to reassess patients who are not benefited with the initial plan of treatment (D-38).

#### Category: Management of LBP

Another overarching theme discussed in the directives is LBP management.

##### Themes

(1) *Self-management* Overwhelmingly, directives encouraged patients to self-manage their pain and this was often discussed as a major component of LBP care. Self-management was often portrayed as a tool that could empower patients in their care (D-4). Many directives focused on helping patients develop and improve self-management skills, providing evidence-based information on right practices and help to avoid dangerous and non-evidence-based options (D-14). A major focus of self-management information is on exercise and lifestyle measures (D-81). The components of self-management advice ranged from ‘ways to stay active’ to ‘Treatment options possible at home’, discussing the role patients can effectively play in management.

(2)* Seeking help from professionals*—Seeking timely and right help from care providers, including medical, psychological, and other allied health services is highlighted in the directives. The directives provided information on the warning signs to be aware of as a sign for the need to seek emergency medical care (D-21).

(3) *Pharmacological therapy*—Apart from listing the pharmacological options available to treat back pain, the directives included some common recommendations such as 1) Start with simple pain killers and topical gels 2) Opioids have a limited role 3) Consult care providers before consuming medicines. Other information discussed in the directives include ways to use pain-medicines effectively and the importance of seeking medical advice regarding medications (D- 41), their benefits and side-effects (D-10).

(4) *Non-pharmacological approaches: (a) Subthemes*

*(1) Exercise*—Most directives explained the importance of exercising, both as a treatment and prevention option (D-4). Some directives also described the diverse types of exercise and how patients could utilise the services providing advice on this (D-5).

*(2) Staying active*—Directives emphasised the importance of staying active, with some of them elaborating on what that meant (D-8). Likewise, the benefits of staying at work and engaging with friends and family even while having pain were highlighted. Staying active was also discussed as a facilitator of recovery (D-35). A few directives elaborated on how remaining active could vary according to individuals’ circumstances (D-46).

*(3) Psychological therapy*—The directives described the various psychological treatment options available to treat the psychological aspects of back pain and the necessity to incorporate them early in treatment (D-28). The directives also highlighted the significance of a positive mindset in back pain management (D- 43).

*(4) Physical therapy—*The directives highlight how physiotherapy, could help manage back pain effectively when combined with pharmacological therapy or as part of rehabilitation and prevention. Some directives elaborated on the limited role of physical therapy options (D-39). Some directives also highlight the different non-pharmacological therapies that can be incorporated into self-management (D-4).

*(5) Surgery—*Another important message given by the directives is that surgical options are required only in rare cases for LBP treatment (D-13).

### Production and publication of directives.

The types of actors who published the directives included universities/academics, charitable not-for-profit organizations, federal/state governments, local public health authorities (Local Health Districts or Networks in Australia), professional associations (for example the Royal Australian College of General Practitioners), private hospitals, and private health care insurers such as Bupa and Hospitals Contribution Fund of Australia.

We identified partnerships between state governments and non-for-profit organisations or hospitals, which combined and produced policy directives.

Still, it was rare to find policy directives that were produced with the involvement of a comprehensive range of stakeholders and authorities from all relevant parts of the health system. Only 5 directives in the repository explicitly mentioned experts and stakeholders that were involved in the development of the document through a steering committee, working party or similar (D-1, D-42, D-44, D-48, D-57).

## Discussion

To our knowledge, our study is the first to consider diverse types of LBP directives developed in Australia. Our primary finding is that there are a variety of directives about LBP care in Australia, developed by a variety of organisations with varied and broad content. However, we also found that the scope and breadth of directives indicate gaps, reflect context specific debate, and suggest a of clear roles and responsibilities for policy.

### Gaps in the landscape

#### Models of care

We found that there is a scarcity of MOC, with only four LBP MOCs developed in Australia that we could identify, with responsibilities for health system innovation and improvement (D-1, D-11, D-32, D-84). Recent addition to the list is a Clinical Care Standard from the Australian Commission on Safety and Quality in Healthcare, released in September 2022, after a gap of 5 years.

The paucity of LBP MOCs is significant considering the number of clinical networks and the work being done in Australian states on MOC development. In WA, following the implementation of health networks in December 2006, more than 50 MOCs across different health conditions and population groups were developed, [8, 13, 21] including musculoskeletal MOCs on inflammatory arthritis (2009) Osteoporosis (2011) and Elective Joint Replacement Service (2010) yet there are none on back pain apart from a Spinal Pain, now considered obsolete by the authors, and not included in our repository [12].Clinical networks have also been functioning in Victoria, South Australia, and Tasmania since 2007–2008 on different specialties and topics, but none are specific to back pain or musculoskeletal health. [21]

Compared to information sheets which provide quick and easy information to clinicians or patients, depending on the targeted audience and guidelines aimed at assisting clinicians to take decisions on specific circumstances, MOCs are broader and are aimed at systems and services rather than at healthcare providers. MOCs contain information on the services delivered in the system, outline best practice care, and incorporate details from and links to guidelines, information sheets and other directives.

An Australian study identified leadership and strategic and operational management as factors boosting the work of clinical networks, which can in turn improve MOC development [14]. As pointed out in studies, what is required to improve MOC development and implementation is more engagement with different stakeholders ranging from health departments, hospital networks, and health districts to regional primary care networks and private sector groups [13, 22], improved leadership, management, and adequate resources and infrastructure. While the challenges to develop and update MOC, including the increased time taken and keeping up the evidence-based nature of MOCs are identified [8], their role in improving musculoskeletal health through recommendations on planning, policy, and financing is significant, and developing and utilising more MOC should be the way forward.

#### Ad hoc directives and lack of updates

Our study found only directives by two organisations (Arthritis Australia and South Australia government-D-25 to D-30) that were updated versions of previous originals. This suggests that directives are being produced ad hoc, or opportunistically when a specific demand arises—rather than as part of a long-term strategy or where there is a clear responsibility. The significance of updating is evident from the examples we found. The version of the information sheet 'Back pain" reviewed in May 2015 mentioned low-evidence based treatment options such as herbal medicines including cayenne, devil's claw, white willow bark and comfrey, which have been omitted in the updated version by Arthritis Australia reviewed in December 2017, which only mentions the evidence-based treatment option of capsaicin patches. [23]

#### Guiding coordinated care

Different initiatives to develop and modify self-management resources aimed at improving musculoskeletal health were undertaken in Australia [24]. A personalized approach in self-management is particularly crucial for those with atypical symptoms and co-morbidities, as some self-management options can have unfavourable effects in these groups [25]. This highlights the need for improved directives concentrating on the importance of tailoring self-management education for different age groups, stages, and types of LBP.

We found a far greater number of information sheets for patients than clinical tools and information sheets for care providers. A notable exception is the work from the South Australian health department where different types of documents for care providers were produced. These included more targeted and specific directives such as diagnostic, imaging and clinical action guides for doctors and analgesia guidelines for nonspecific low back pain and neuropathic pain of spinal origin (D-25 to D-30). This may indicate a greater perceived need for patient-oriented information; or the challenges of providing comprehensive guidance for individual clinicians in a complex healthcare system for a condition that requires multidisciplinary care delivery. We found few directives that integrated pathways and flowcharts, considering coordinated multidisciplinary care delivery is the ideal model for LBP care provision [7].Such directives can help care providers to efficiently manage LBP patients while promoting personalisation of their management approach according to patients’ clinical profile and making the right care-seeking easier for patients.

### Relevance of the identified themes and subthemes in the Australian context

Overall, directives in our repository cover a wide breadth of content ranging from defining low back pain in terms of types and causes, standards for clinical encounters and management of LBP. However, the themes covered also indicate a context determined focus reflecting current LBP care research and debate in Australia. For example, **t**he significance of history taking, and examination [26] is a key message in the directives for both patients and care providers. This corelates with studies that reported that the completion rate by GPs for certain crucial details in history and examination to be low. [25]. Directives that include details of diagnostic triage through red and yellow flag signs can be seen in the context of Australian studies reporting an undesirable percentage of patients being examined for signs and symptoms closely related to LBP aetiology [25]. However, care providers also need to be cautious while using the triage methods with unproven accuracy [27, 28] and not over-depend on them, as systematic reviews warn about their misleading nature [27].

Australian studies have also reported a higher percentage of unnecessary imaging [9] and studies on the role of overdiagnosis leaflets [29], and emergency department point of care tools [30] in delaying unnecessary imaging in Australia, underline the significance of directives in reminding patients and care providers of this issue.

### Fragmented roles and responsibilities

The diversity in actors that produces LBP directives is reflective of a complex health system as well as opaque roles and responsibilities within it. The production of directives from different states and different levels of organisations, within the same state, is a proof of work in silos, which can be considered as waste of resources. Despite the aforementioned existence of government agencies that coordinate clinical networks at state levels, as well the existence of an Australian National Strategic Action Plan for Pain Management from the central (federal) government; LBP directives are produced at multiple levels from government, non-government, academic and member-based organisations. This reflects a lack of lines of authority for quality improvement in health care, as well as limited dissemination, implementation and consequently adoption of existing LBP directives in setting beyond the immediate target audience.

### Limitations and scope for future studies

Our search for LBP directives focused on publicly available documents, snowballing and directives known in a network of experts. However, our repository may miss directives that are produced and distributed in smaller settings, such as hospital wards or private sector clinics. We also did not include guidelines for purchase from private providers.

Dimensions of directives such as comprehensiveness, accuracy of information and consistency of information between directives, were not formally assessed due to the heterogeneity in type and scope of the included directives. We propose further research qualitative research to understand the context in which directives are produced and how they are disseminated and implemented. As most of the directives are information sheets, it would also be relevant to undertake a qualitative study on the readability and usability of information sheets. Studies are also required to assess the utility of directives among different types of healthcare providers and the requirement for more and updated directives, which can be confirmed through qualitative studies involving interviews with care providers.

## Conclusion

Directives have the potential to inform practice and to contribute to reducing evidence-policy-practice discordance. While our content analysis demonstrated that a range of directives exist across Australia, the evidence base for many was not apparent. While there has been increasing attention given to models of care, this is not yet reflected in directives, which generally focus on more specific elements of LBP care at the individual patient and practitioner level (Table 2).

**Table 2:** List of recommendations

1. A consistent and coordinated approach to low back pain directives should be used to address the siloed approaches of directive development and reduce potential overlap and gaps	
2. The development of more Models of Care would move policy directives beyond clinical guidance towards whole of systems requirements for improved consumer journeys within the health system	
3. Low back pain directives should meet minimum standards of data documentation, including date of development, due date for revision or expiry, intended audience, relationships to other policy documents and intended audience and use	
4. Patient information sheets should be evaluated for their evidence-based nature and quality, prior to release	
5. Investment in dissemination and awareness strategies of policy directives could reduce instances of ad hoc directives being developed to fill perceived gaps	

This qualitative content analysis highlights the paucity of comprehensive MOC and the variety of content in LBP directives, the knowledge of which is fundamental to develop improved directives, that can promise improved care. The quality and implicit purpose of information sheets vary drastically. This should be preceded by qualitative studies understanding the needs of healthcare providers concerning the directives required by them for use in practice and the needs of patients. There is a need for clearer, easily accessible trustworthy policy directives that are regularly reviewed and that meet the needs of care providers, and information websites need to be evaluated regularly for their evidence-based nature and quality.

## Supplementary Information

**Additional file 1.** List of directives.

**Additional file 2.** Excerpts from directives

## Data Availability

The datasets used and/or analysed during the current study are available from the corresponding author on reasonable request.
