# Supplementary material for: What guides back pain care? A content analysis of low back pain directives in the Australian context
Source: Health Res Policy Syst. 2023 Jun 13;21:49. doi: 10.1186/s12961-023-00997-5 (PMC10262351; doi:10.1186/s12961-023-00997-5)
Supplement: Supplementary file 1 — Additional file 1. List of directives. [file 12961_2023_997_MOESM1_ESM.docx]

| **Document** | **Type of document** | **Year** | **Directive number** | **URL** |
| --- | --- | --- | --- | --- |
| ACUTE LOW BACK PAIN FLOWCHART | Clinical tool-Flow chart | 2017 | D-2 | <https://aci.health.nsw.gov.au/__data/assets/pdf_file/0005/346253/ACI-D16-257-Acute-Low-Back-Pain-Flowchart-January-20175.pdf> |
| Arthritis Information Sheet-Low Back Pain | Information sheet-Patients | 2007 | D-10 | <https://arthritisaustralia.com.au/wordpress/wp-content/uploads/2017/09/Back-pain.pdf> |
| Analgesia guideline – non- specific low back pain | Clinical tool-Management tool | 2011 | D-29 | <https://www.sahealth.sa.gov.au/wps/wcm/connect/public+content/sa+health+internet/clinical+resources/clinical+programs+and+practice+guidelines/medical+conditions/orthopaedics/lumbar+disorders/clinical+decision+tools+on+lumbar+disorders> |
| Analgesia guideline – neuropathic pain of spinal origin (eg lumbar radiculopathy) | Clinical tool-Management tool | 2011 | D-30 | <https://www.sahealth.sa.gov.au/wps/wcm/connect/public+content/sa+health+internet/clinical+resources/clinical+programs+and+practice+guidelines/medical+conditions/orthopaedics/lumbar+disorders/clinical+decision+tools+on+lumbar+disorders> |
| Acute back pain with red flags | Information sheet-Care providers | 2019 | D-34 |  |
| Acute low back pain- What you can do to help yourself get better (Self-Management diary) | Information sheet-Patients | 2016 | D-40 |  |
| Advice to stay active for people with low back pain  Intervention | Information sheet-Patients | 2013-2016 | D-46 | <https://www.racgp.org.au/clinical-resources/clinical-guidelines/handi/handi-interventions/musculoskeletal/advice-to-stay-active-for-people-with-back-pain> |
| Acute short term Low back pain | Information sheet-Patients |  | D-66 | <https://www.choosingwisely.org.au/resources/consumers-and-carers/acute-short-term-low-back-pain#:~:text=Stay%20active%20and%20keep%20moving,30%20minutes%20at%20a%20time.> |
| Acute low back pain | Information sheet-Patients |  | D-68 | <https://aci.health.nsw.gov.au/networks/eci/clinical/clinical-tools/orthopaedic-and-musculoskeletal/acute-low-back-pain> |
| Advice for managing low back pain | Information sheet-Patients | 2011 | D-69 | ManagingLowBackPain-RAH-AlliedHealth-120123.pdf (sahealth.sa.gov.au) |
| Advice for managing sciatica | Information sheet-Patients | 2011 | D-70 | ManagingSciatica-RAH-AlliedHealth-120123.pdf (sahealth.sa.gov.au) |
| Acute back pain without leg symptoms | Information sheet-care providers | 2019 | D-74 |  |
| Acute back pain with leg symptoms | Information sheet-care providers | 2019 | D-75 |  |
| Back pain (Musculoskeletal Australia) | Information sheet-Patients | 2019 | D-4 | <https://www.msk.org.au/back-pain/> |
| Back Pain (Better health channel) | Information sheet-Patients | 2021 | D-13 | <https://www.betterhealth.vic.gov.au/health/conditionsandtreatments/Back-pain> |
| Back Pain, Emergency Department fact sheets | Information sheet-Patients |  | D-20 | <https://www.health.qld.gov.au/__data/assets/pdf_file/0020/621164/ed-back_pain.pdf> |
| Back pain (Pharmaceutical society of Australia) | Information sheet-Patients | 2011 | D-36 | <http://www.hardingspharmacy.com.au/wp-content/uploads/2016/08/BackPain-web.pdf> |
| BEATING LOWER BACK PAIN | Information sheet-Patients | 2014 | D-37 | <https://www.hcf.com.au/health-agenda/health-care/common-conditions/back-on-track> |
| Best practice care for people with acute low back pain- Consumer information | Information sheet-Patients | 2019 | D-38 | <https://aci.health.nsw.gov.au/resources/pain-management/acute-sub-acute-pain/albp-model> |
| Back pain (BUPA) | Information sheet-Patients | 2019 | D-39 | <https://www.bupa.com.au/healthlink/conditions-and-treatments/common-illness-and-conditions/clinical-health-information/back-pain> |
| Back pain (Health Direct) | Information sheet-Patients | 2020 | D-48 | <https://www.healthdirect.gov.au/back-pain> |
| BACK SURVEY | Survey and questionnaire for patients |  | D-64 | <https://metrosouth.health.qld.gov.au/referrals/orthopaedics/spinal-surgical> |
| Back pain-Disc problems | Information sheet-Patients | 2020 | D-71 | <https://www.betterhealth.vic.gov.au/health/conditionsandtreatments/back-pain-disc-problems> |
| Back pain in children | Information sheet-Patients | 2019 | D-72 | Back pain in children - Better Health Channel |
| Back pain assessment Clinic- Patient information brochure | Information sheet-Patients |  | D-81 |  |
| Back Assessment Clinic (Non-Emergency Low Back Pain) | Referral criteria |  | D-83 |  |
| Consumer’s guide to managing back pain | Information sheet-Patients |  | D-7 | <https://painhealth.csse.uwa.edu.au/wp-content/uploads/2016/04/Consumers-Guide-for-Low-back-Pain.pdf> |
| Clinical guidelines for the Queensland workers' compensation scheme | Guidelines | 2008 | D-12 | <https://myosh.com/wp-content/uploads/2017/11/back1-1.pdf> |
| Clinical action guide | Clinical tool-Management tool | 2011 | D-28 | <https://www.sahealth.sa.gov.au/wps/wcm/connect/public+content/sa+health+internet/clinical+resources/clinical+programs+and+practice+guidelines/medical+conditions/orthopaedics/lumbar+disorders/clinical+decision+tools+on+lumbar+disorders> |
| Diagnostic Imaging Pathways – Low Back Pain | Guidelines | 2013 | D-17 | <http://www.imagingpathways.health.wa.gov.au/index.php/imaging-pathways/musculoskeletal-trauma/musculoskeletal/low-back-pain> |
| Diagnostic Imaging Clinical Committee Low Back Pain Report Taskforce Findings | Taskforce report |  | D-61 | <https://www1.health.gov.au/internet/main/publishing.nsf/Content/E1FEC9FFE18698C0CA25801800184170/$File/MBS-Review-Taskforce-Recommendations-DI-Low-Back-Pain-Report.docx> |
| Expanding community workforce capacity to  deliver multidisciplinary non-surgical  management of back pain in community based settings | Model of care | 2015 | D-11 | <https://collaborative.org.au/wp-content/uploads/2017/02/12.-Back-pain-Report.pdf> |
| Exercise: Chronic low back pain | Information sheet-Care providers | 2016 | D-44 |  |
| Exercise for acute lower back pain | Information sheet-care providers |  | D-49 | <https://www.racgp.org.au/clinical-resources/clinical-guidelines/handi/handi-interventions/exercise/exercise-for-acute-lower-back-pain> |
| Effective ways to help back pain | Information sheet-Patients |  | D-67 |  |
| First Report from the Diagnostic Imaging Clinical Committee ‐ Low Back Pain | Committee report | 2016 | D-15 | <https://www1.health.gov.au/internet/main/publishing.nsf/Content/mbsr-first-report-diagnostic-imaging-clinical-committee-low-back-pain> |
| Improving care of patients with back and neck pain | Model of care | 2017 | D-32 | <https://clinicalexcellence.qld.gov.au/improvement-exchange/back-neck-pain> |
| Is Paracetamol effective for low back pain | Information sheet-patients | 2014 | D-58 | <https://www.nps.org.au/news/is-paracetamol-effective-for-low-back-pain#:~:text=An%20Australian%20study%20has%20found,for%20acute%20low%20back%20pain.> |
| Information for Consumers - Low Back Pain (Acute) | Information sheet-Patients | 2017 | D-73 | <http://www.imagingpathways.health.wa.gov.au/index.php/consumer-info/imaging-pathways/acute-low-back-pain> |
| Low back pain clinical care standard | Model of care | 2022 | D-84 | <https://www.safetyandquality.gov.au/standards/clinical-care-standards/low-back-pain-clinical-care-standard> |
| Low back pain: Promoting wise healthcare | Report on value care |  | D-6 | <https://painhealth.csse.uwa.edu.au/wp-content/uploads/2016/04/painHEALTH-low-back-pain-promoting-wise-healthcare.pdf> |
| Low back pain (Therapeutic guidelines) | Information sheet-Patients | 2020 | D-8 |  |
| Low back pain-Do you need a scan? | Information sheet-patients | 2018 | D-9 | <https://www.nps.org.au/assets/81ab7562891245b2-5948693db67c-NPS2060_LBP_FactSheet_v7-as-accessible.pdf> |
| Low back pain-What you can do? | Information sheet-patients |  | D-16 | <https://www.aci.health.nsw.gov.au/__data/assets/pdf_file/0019/216307/Pain_Management_TAG.pdf> |
| LOW BACK PAIN MODULE | Information sheet-care providers | 2015 | D-18 | <https://www.notredame.edu.au/__data/assets/pdf_file/0009/3033/Low-back-pain-module-V1.1.pdf> |
| Low back pain (Safer care Victoria) | Information sheet-patients | 2019 | D-19 | <https://www.bettersafercare.vic.gov.au/clinical-guidance/emergency/low-back-pain> |
| Lumbar disorders: physical examination of the lumbar spine | Clinical tool-examination tool | 2011 | D-25 | <https://www.sahealth.sa.gov.au/wps/wcm/connect/public+content/sa+health+internet/clinical+resources/clinical+programs+and+practice+guidelines/medical+conditions/orthopaedics/lumbar+disorders/clinical+decision+tools+on+lumbar+disorders> |
| Lumbar disorders – a diagnostic guide | Clinical tool-diagnostic tool | 2011 | D-26 | <https://www.sahealth.sa.gov.au/wps/wcm/connect/public+content/sa+health+internet/clinical+resources/clinical+programs+and+practice+guidelines/medical+conditions/orthopaedics/lumbar+disorders/clinical+decision+tools+on+lumbar+disorders> |
| Lumbar disorders – imaging guideline | Clinical tool-diagnostic tool | 2011 | D-27 | <https://www.sahealth.sa.gov.au/wps/wcm/connect/public+content/sa+health+internet/clinical+resources/clinical+programs+and+practice+guidelines/medical+conditions/orthopaedics/lumbar+disorders/clinical+decision+tools+on+lumbar+disorders> |
| Low back pain-Case study for health professionals | Clinical tool-case study |  | D-33 | <https://www.nps.org.au/professionals/low-back-pain> |
| Low back pain recovery plan | Information sheet -patients | 2018 | D-35 | <https://www.nps.org.au/assets/7d7d2d33725d9da3-adb894d51a10-NPS-MedicineWise-Low-back-pain-recovery-plan.PDF> |
| Low back pain (Department of health, WA) | Information sheet-Patients |  | D-42 | <https://healthywa.wa.gov.au/Articles/J_M/Low-back-pain> |
| Low back pain (Pain health) | Information sheet-patients | 2020 | D-53 | <https://painhealth.csse.uwa.edu.au/pain-module/low-back-pain/> |
| Low back pain (General Practice Supervisors Australia) | Information sheet-GP supervisors |  | D-55 | <http://gpsupervisorsaustralia.org.au/wp-content/uploads/2016/12/Teaching-aid_back-pain_V5.pdf> |
| Low back pain (Exercise and Sports Science Australia) | Information sheet-patients |  | D-57 | <https://exerciseright.com.au/lower-back-pain/> |
| Lumbar disorders: diagnostic imaging in low back pain | Clinical tool-Diagnostic tool | 2011 | D-60 | <https://www.sahealth.sa.gov.au/wps/wcm/connect/public+content/sa+health+internet/clinical+resources/clinical+programs+and+practice+guidelines/medical+conditions/orthopaedics/lumbar+disorders/clinical+resources+for+lumbar+disorders/spinal+imaging+recommendations> |
| Low Back Pain Clinical Care Standard Public Consultation | Public enquiry | 2021 | D-79 |  |
| Model of Care- Management of people with  acute low back pain- Musculoskeletal network | Model of care | 2016 | D-1 | <https://aci.health.nsw.gov.au/resources/musculoskeletal/management-of-people-with-acute-low-back-pain/albp-model>https://aci.health.nsw.gov.au/__data/assets/pdf_file/0007/336688/acute-low-back-pain-moc.pdf |
| Management of acute non-specific low back pain  Information for health professionals | Information sheet-Patients | 2016 | D-14 | <https://www2.health.vic.gov.au/-/media/health/files/collections/policies-and-guidelines/safe-opiod-use/management-of-acute-non-specific-low-back-pain---for-health-professionals.pdf> |
| Managing a first or acute episode of low back pain | Information sheet-patients | 2014 | D-21 | <http://exerciseismedicine.com.au/wp-content/uploads/2018/06/2014-Low-Back-Pain-FULL.pdf> |
| Managing low back pain Information for patients | Information sheet-patients | 2019 | D-22 | <https://aci.health.nsw.gov.au/resources/pain-management/acute-sub-acute-pain/albp-model> |
| Managing low back pain and sciatica | Information sheet-patients |  | D-41 | Managing low back pain and sciatica \| SA Health |
| Mindfulness and cognitive behavioural therapy:  chronic low back pain | Information sheet-providers | 2017 | D-47 | <https://www.racgp.org.au/clinical-resources/clinical-guidelines/handi/handi-interventions/cogntive-and-behavioural-therapies/mindfulness-and-cbt-for-chronic-low-back-pain> |
| Managing back pain (BUPA) | Information sheet-patients | 2020 | D-50 | <https://www.bupa.com.au/healthlink/conditions-and-treatments/common-illness-and-conditions/clinical-health-information/back-pain> |
| Managing your back pain (Tasmanian health service) | Information sheet-patients |  | D-80 |  |
| Non-Emergency Low Back Pain | Referral criteria |  | D-82 |  |
| Patient factsheet- acute low back pain | Information sheet-patients | 2014 | D-3 | <https://www.healthshare.com.au/factsheets/12323-acute-low-back-pain/> |
| PRESCRIBING GUIDELINES FOR PRIMARY CARE CLINICIANS- LOW Back Pain | Prescribing guidelines | 2002 | D-5 | <https://www.nswtag.org.au/practical-guidance/> |
| Paediatric Back Pain | Referral criteria |  | D-78 |  |
| Recommendations from the Faculty of Pain Medicine, ANZCA on chronic pain, neuropathic pain and low back pain. | Information sheet-providers | 2018 | D-52 | <https://www.choosingwisely.org.au/recommendations/fpm> |
| Recommendations from the Australian Physiotherapy Association on low back pain | Information sheet-providers | 2016 | D-54 | <https://www.choosingwisely.org.au/recommendations/apa1> |
| Recommendations RANZCR | Information sheet-providers | 2015 | D-56 | <https://www.choosingwisely.org.au/recommendations/ranzcr> |
| Staying active for acute low back pain | Information sheet- Patients | 2020 | D-45 | <https://www.racgp.org.au/clinical-resources/clinical-guidelines/handi/patient-resources/musculoskeletal/staying-active-for-acute-low-back-pain> |
| Scans and low back pain | Information sheet-Patients | 2011 | D-59 | <https://www.sahealth.sa.gov.au/wps/wcm/connect/public+content/sa+health+internet/conditions/pain/low+back+pain/scans+and+low+back+pain> |
| Spine, Neck, Back Pain | Information sheet- Care providers |  | D-62 |  |
| Smoking and low back pain | Information sheet-Patients | 2011 | D-63 | <https://www.sahealth.sa.gov.au/wps/wcm/connect/public+content/sa+health+internet/resources/smoking+and+low+back+pain> |
| Spinal Surgical Service Referral Form | Referral form |  | D-65 | <https://metrosouth.health.qld.gov.au/referrals/orthopaedics/spinal-surgical> |
| Spine (Orthopaedics) | Referral criteria |  | D-76 |  |
| Spine (Neurosurgery) | Referral criteria |  | D-77 |  |
| Spinal Referral Questionnaire | Questionnaire | 2017 | D-31 | <https://www.ourphn.org.au/wp-content/uploads/2017/05/spinal-questionnaire.pdf> |
| Triage and referral guideline – the assessment of lumbar disorders | Referral Guidelines- Clinicians | 2011 | D-23 | https://www.sahealth.sa.gov.au/wps/wcm/connect/public+content/sa+health+internet/clinical+resources/clinical+programs+and+practice+guidelines/medical+conditions/orthopaedics/lumbar+disorders/clinical+decision+tools+on+lumbar+disorders |
| Understanding my low back pain AND WHETHER I NEED IMAGING | Information sheet-Patients |  | D-43 | <https://www.mq.edu.au/__data/assets/pdf_file/0012/1023123/Digital-booklet.pdf> |
| 10-things-you-need-to-know-about-low-back-pain | Information sheet-patients | 2018 | D-24 | <https://www.nps.org.au/consumers/10-things-you-need-to-know-about-low-back-pain> |
| 5 QUESTIONS TO ASK ABOUT USING OPIOIDS  FOR BACK PAIN OR OSTEOARTHRITIS | Information sheet-patients |  | D-51 | <https://www.nps.org.au/assets/5-questions-to-ask-about-using-opioids-for-back-pain-and-OA-final-resource.pdf> |
