# Supplementary material for: What guides back pain care? A content analysis of low back pain directives in the Australian context
Source: Health Res Policy Syst. 2023 Jun 13;21:49. doi: 10.1186/s12961-023-00997-5 (PMC10262351; doi:10.1186/s12961-023-00997-5)
Supplement: Supplementary file 2 — Additional file 2. Excerpts from directives [file 12961_2023_997_MOESM2_ESM.docx]

**Excerpts from directives in the theme description**

**|3.2.1 |Category: Definitions, causes and descriptions of LBP as a condition**

**3.2.1.a |Themes:**

***1)Definitions and anatomy***

**D-3**

“The back is made up of the spinal cord and its branches (nerve roots) which are protected by relatively small bones (vertebrae)”

***2)Causes of LBP***

**D-13**

“Common causes of back pain include repetitive or heavy lifting, sudden awkward movement not getting enough regular physical activity, poor posture, being overweight”

***3)Types of LBP***

***D-14***

“The term ‘non-specific’ means that the anatomical structure causing the pain can’t be identified because there are no tests available that can achieve this”

**3.2.2 |Category: Standards for clinical encounters**

**3.2.2.a |Themes**

***I)Person-centred care***

**D-14**

“Normal activity advice varies for each individual patient. For an office worker it may mean going to work but avoiding sitting for prolonged periods. For an athlete it may mean only doing light training”

**D-1**

“The clinical care providers must consider the individual’s preferences after discussing and disclosing the risks, benefits, safety and relative lack of long-term effectiveness of treatment options for ALCP”

***2)Multidisciplinary care***

***D-32***

“A single screening and triage hub will review all new referrals and those waiting to see a specialist. They will aim to connect patients to appropriate services as rapidly as possible, form alliances and improve communication between healthcare professionals”

D-13

"Multidisciplinary pain management involving a team that often includes specialist pain physicians, physiotherapists, occupational therapists and psychologists some medications”

***3)Patient education***

D-12

“Patient education regarding healthy lifestyle and general aerobic fitness with emphasis on patient responsibility for good back care, workplace ergonomics and home self-care”

D-38

“Back pain education: Your healthcare professional will have a one-on-one discussion with you about your pain and self- management strategies”

D-6

“Accurate education about your back pain that fosters a positive mindset”

***4)Appropriate history and examination***

***D-37***

“Your healthcare professional will assess your acute low back pain by asking about your symptoms and your medical history and performing a physical examination”

**D-41**

“Your healthcare professional can assess the risk of serious disorders causing low back pain. They can guide you on the best treatment and management of your pain”

***5)Investigations (e.g., imaging)***

**D-9**

“Among 100 people who have low back pain: The majority, about 90, have low back pain for which a cause can't be found. Less than 1 person has a serious cause, may need a scan”

**D-21**

“Lower back x-rays and other scans usually do not show the cause of pain. They do not change how your pain is treated and expose you to radiation”

***6)Risk stratification***

***7)Reassurance***

***D-10***

"You should talk to your doctor or other health professional if your pain is bothering you. They may check for any serious medical problems that could be causing your pain, but these are rare".

**D-20**

“Most back pain tends to improve within a few days to a few weeks, and a therapist such as a physiotherapist, chiropractor or osteopath can assist with exercises, advice and treatment”

***D-10***

“The sooner you get back to your normal activities the sooner you will recover from a bout of back pain”

**D-1**

“Reassure and explain that back pain is a symptom and that in most situations it does not indicate serious disease or impending long-term disability”

***8)Referral***

***D-38***

“It usually takes up to 12 weeks to recover from acute low back pain. If you have not recovered after 12 weeks, you should be reviewed again by your doctor, who will consider if specialist musculoskeletal assessment and treatment are required”

**D-82**

“In the South, patients with non-emergency lower back pain with or without lower limb neurological signs and symptoms requiring specialist assessment and management, including surgical opinion, should be referred to the Back Assessment Clinic”

**D-81**

“co-ordination of referrals to other health professionals such as radiology (MRI / CT etc), physiotherapy, pain management”

***9)Follow up and reassessment***

***D-8***

“To reassure patients and aid confidence in self-management, schedule a review appointment at 4 to 6 weeks”

**D-38**

“People with acute low back pain should be reviewed regularly to make sure they are recovering and feel supported during this episode of pain. If you are not improving, your treatment plan will be changed accordingly”

**3 |Category: Management of LBP**

**3.2.3.a |Themes**

**1) *Self-management***  ***D-4***

“Knowing as much as possible about your condition means that you can make informed decisions about your healthcare and play an active role in the management of your condition. Learn ways to manage pain – there are many strategies you can use to deal with pain”

**D-14**

“Self-management is based on clear information and advice about the nature of non-specific LBP and encouragement to stay active and continue doing normal activities. This includes reassuring patients that they don’t have serious damage or disease and informing them that the evidence shows that improvement is more likely when they do normal activities rather than rest in bed”

**D-81**

“With the right information, support and treatment, most people can manage their own back pain”

***2) Seeking help from professionals***

***D-21***

“You should seek advice from a health care practitioner as soon as possible if your back pain occurs after a traumatic event, such as a car accident or a fall; does not reduce or change, no matter how you move or change position; accompanies numbness, tingling, or ‘pins and needles’ in your bottom, legs or feet; or accompanies changes to your bowel or bladder control”

**3) *Pharmacological therapy***

***D-41***

“Taking pain relief regularly is often important to help you to keep moving”

**D-10**

“Medicines: non-steroidal anti-inflammatories (NSAIDs) may help some people”

***4) Non-pharmacological approaches***

***4.a   |Subthemes***

***1)Exercise***

***D-4***

“exercise regularly – exercise is key to a healthy back. It will improve your posture and increase muscle support of the spine"

**D-5**

"Facilitate active rehabilitation program e.g., attendance at ‘back school’ in the workplace”

***2)Staying active***

**D-8**

"Encourage patients with nonspecific low back pain to stay active, including adopting normal movement and physical function as much as possible, and continuing or returning to work”

**D-35**

"Staying active reduces pain and time off work and speeds up your recovery’’

**D-46**

“It means different things to different patients. For an office worker it might mean avoiding sitting for long periods and using a sit-stand desk, while for an athlete it might mean light training”

***3)Psychological therapy***

**D-28**

“Referral to a psychologist may be indicated if yellow flags are predominant, Cognitive behavioural therapy is a recommended treatment option in cases of persistent pain”

**D-43**

"Managing these feelings might help your low back pain. Try to stay positive and take steps towards a healthier lifestyle”

***4)Physical therapy***

**D-39**

“Manual (physical) therapies may be recommended as part of a treatment program for back pain. This may involve things like exercises, posture advice or massage”

**D-4**

“Apply hot and cold packs applied to the area of pain may be helpful in relieving pain temporarily”

***5)Surgery***

***D-13***

“Surgery is rarely needed for back pain unless a more serious issue or condition is causing your back pain, or if nerve compression is present”
